# Obesogens beyond Vertebrates: Lipid Perturbation by Tributyltin in the Crustacean *Daphnia magna*

**DOI:** 10.1289/ehp.1409163

**Published:** 2015-03-24

**Authors:** Rita Jordão, Josefina Casas, Gemma Fabrias, Bruno Campos, Benjamín Piña, Marco F.L. Lemos, Amadeu M.V.M. Soares, Romà Tauler, Carlos Barata

**Affiliations:** 1 Department of Environmental Chemistry, Institute of Environmental Assessment and Water Research (IDAEA), Spanish Research Council (IDAEA, CSIC), Barcelona, Spain; 2 Centre for Environmental and Marine Studies (CESAM), Department of Biology, University of Aveiro, Aveiro, Portugal; 3 School of Tourism and Maritime Technology (ESTM), Marine Resources Research Group (GIRM), Polytechnic Institute of Leiria, Peniche, Portugal; 4 Department of Biomedicinal Chemistry, Institute for Advanced Chemistry of Catalonia, Spanish Research Council (IQAC-CSIC), Barcelona, Spain

## Abstract

**Background:**

The analysis of obesogenic effects in invertebrates is limited by our poor knowledge of the regulatory pathways of lipid metabolism. Recent data from the crustacean *Daphnia magna* points to three signaling hormonal pathways related to the molting and reproductive cycles [retinoic X receptor (RXR), juvenile hormone (JH), and ecdysone] as putative targets for exogenous obesogens.

**Objective:**

The present study addresses the disruptive effects of the model obesogen tributyltin (TBT) on the lipid homeostasis in *Daphnia* during the molting and reproductive cycle, its genetic control, and health consequences of its disruption.

**Methods:**

*D. magna* individuals were exposed to low and high levels of TBT. Reproductive effects were assessed by Life History analysis methods. Quantitative and qualitative changes in lipid droplets during molting and the reproductive cycle were studied using Nile red staining. Lipid composition and dynamics were analyzed by ultra-performance liquid chromatography coupled to a time-of-flight mass spectrometer. Relative abundances of mRNA from different genes related to RXR, ecdysone, and JH signaling pathways were studied by qRT-PCR.

**Results and Conclusions:**

TBT disrupted the dynamics of neutral lipids, impairing the transfer of triacylglycerols to eggs and hence promoting their accumulation in adult individuals. TBT’s disruptive effects translated into a lower fitness for offspring and adults. Co-regulation of gene transcripts suggests that TBT activates the ecdysone, JH, and RXR receptor signaling pathways, presumably through the already proposed interaction with RXR. These findings indicate the presence of obesogenic effects in a nonvertebrate species.

**Citation:**

Jordão R, Casas J, Fabrias G, Campos B, Piña B, Lemos MF, Soares AM, Tauler R, Barata C. 2015. Obesogens beyond vertebrates: lipid perturbation by tributyltin in the crustacean *Daphnia magna.* Environ Health Perspect 123:813–819; http://dx.doi.org/10.1289/ehp.1409163

## Introduction

In mammals, improper control of lipid homeostasis can result in serious health problems, such as obesity, increased risk of coronary artery diseases, diabetes, and related detrimental effects, such as hypertension and lipidemia (Grün and Blumberg 2006; Sharma and Staels 2007). The nuclear receptor peroxisome proliferator-activated receptor γ (PPARγ), together with its heterodimeric partner retinoid X receptor (RXR), are master regulators of adipocyte differentiation, being involved in the regulation of food intake, metabolic efficiency, and energy storage (Santos et al. 2012). Organotins such as tributyltin (TBT) are high-affinity ligands of both RXRs and PPARγ (Santos et al. 2012). Organotins stimulate cell differentiation and the expression of adipocyte marker genes, elevate lipid accumulation in several tissues of mice, and increase adipocytes in zebra fish juveniles (Santos et al. 2012). Although PPAR has not been described outside deuterostomes, RXR is ubiquitous within metazoans. Thus, the taxonomic scope for organotin-mediated lipid homeostasis disruption may be wider than initially anticipated.

Recently, Wang and colleagues (Wang and LeBlanc 2009; Wang et al. 2011) showed that in the cladoceran crustacean species *Daphnia magna*, RXR is activated by TBT and elicits toxicity by interfering with the ecdysone signaling pathway. Nevertheless, there are no reported results on whether or not lipid profiles are impacted by TBT. In *D. magna* molting, growth and reproductive functions modulate the quantity and fate of storage lipids, mainly triacylglycerols located in spherical lipid droplets inside fat cells scattered throughout the animal hemocoel (Tessier and Goulden 1982; Zaffagini and Zeni 1986). Lipid droplets and/or triacylglycerid levels increase during the intermolt cycle and are reduced after being allocated to the molt in juvenile stages or to the molt and egg formation in adult stages (Tessier and Goulden 1982). In adult *D. magna* reared under high-food-ration conditions, triacylglycerols may increase from 3- to 6-fold during the intermolt cycle (Goulden and Place 1990). These accumulated lipids are subsequently utilized for reproduction and growth (Tessier and Goulden 1982). Storage lipids are related to starvation tolerance. Neonates with high maternal storage lipids survive longer than those with lower levels (Tessier et al. 1983).

Ecdysteroids and juvenoids have a major role in regulating molting, growth, and reproduction in crustaceans. Ecdsyteroids, such as ecdysone, exhert their effects through the interaction with the ecdysone receptor (EcR), known to heterodimerize with RXR and to bind to the promoters of ecdysone-regulated genes (LeBlanc 2007; Wang and LeBlanc 2009). EcR regulates the expression of a number of genes such as *HR3*, *HR78*, and *HR96* (LeBlanc 2007). This regulatory activity is controlled by RXR (LeBlanc 2007; Mu and LeBlanc 2004). Recent findings indicate that the juvenile hormone receptor in *Daphnia* is a complex of two nuclear proteins of the bHLH-PAS family of transcription factors: the methoprene-tolerant receptor (MET) and steroid receptor coactivator (SRC) protein (Miyakawa et al. 2013). Juvenoids promote expression of hemoglobin genes, such as *Hb2,* and sex-determining genes in the latter stages of ovarian oocyte maturation and supress *HR38* in *Daphnia* (LeBlanc 2007). TBT acts as a disruptor of this regulatory pathway because the EcR:RXR heterodimer can be activated by TBT and juvenoids under the presence of ecdysteroids (Wang and LeBlanc 2009). Thus, it is plausible that TBT may alter lipid homeostasis in the crustacean *D. magna* by interacting with ecdysone and/or the juvenile signaling pathway.

In the present study, we used Nile red to quantify the amount of lipid droplets in whole *D. magna* individuals during the first reproductive instar (the so-called adolescent instar) across food levels and under exposure to TBT. During the adolescent instar, *Daphnia* females provision the first clutch of eggs (Barata and Baird 1998). Life-history effects of the disruption of this provision process by TBT were analyzed by testing the tolerance to starvation and life-history performance of adult females exposed during the adolescent instar and of the neonates hatched from eggs provisioned under organotin exposure. Effects of TBT on the lipid profiles in adults and eggs were assessed by lipidomic nontarget analysis using ultra-performance liquid chromatography coupled to a time-of-flight mass spectrometer (UPLC-TOF) (Gorrochategui et al. 2014). To identify hormonal signaling pathways affected by TBT, we studied changes in mRNA abundance on genes related to ecdysone (*EcRB, HR3, HR38,* and *Neverland*), methyl farnesoate (*Hb2, SCR,* and *MET*), and retinoic acid receptor (*RXR*) signaling pathways (LeBlanc 2007).

## Materials and Methods

*Chemicals*. Tributyltin chloride (TBT; CAS No. 1461-22-9) and Nile red (CAS No. 7385-67-3) were purchased from Sigma-Aldrich, and lipid standards were from Advanti Polar Lipids. All other chemicals were analytical grade and were obtained from Merck.

*TBT treatments*. TBT was dissolved in acetone; the same amount of acetone (< 0.1 mL/L) was used for a solvent control and in all experimental treatments except in the untreated control (control) to account for any carrier effect. Actual TBT concentrations in test solutions were measured as total tin using a Perkin-Elmer Elan 6000 inductively coupled plasma mass spectrometer (ICP-MS) (Barata et al. 2005), and were confirmed to be within 10% of nominal concentrations (0.036 and 0.36 μg/L for 0.1 and 1 μg/L doses, respectively).

*Experimental animals*. All experiments were performed using the well-characterized clone F of *D. magna* maintained indefinitely as pure parthenogenetic cultures (Barata and Baird 1998). Individual cultures were maintained in 100 mL of ASTM hard synthetic water at low and high food-ration levels (*Chlorella vulgaris*, 1 × 10^5^ and 5 × 10^5^ cells/mL, respectively), as described by Barata and Baird (1998).

*Experimental design*. Experiments were initiated with newborn neonates < 4–8 hr old obtained from synchronized females cultured individually at high food-ration levels. Groups of five neonates (F0) were reared in 150 mL of ASTM hard water under high food-ration conditions until the end of the third juvenile instar (about 4–8 hr before molting for the third time). At this point, juveniles were used in three sets of experiments using two TBT treatments, 0.1 μg/L (low; TBT L) and 1 μg/L (high; TBT H). Five to 10 replicates per treatment were used.

The first experiment studied effects of exposure to TBT during the adolescent instar (i.e., 3 days) on the life history of these females (F0) through five consecutive clutches. Their first clutch of neonates, exposed during the egg-provisioning stage (F1) was similarly studied during four consecutive clutches. Following exposure to TBT, F1 females were cultured individually under high food conditions without TBT, and their growth and reproduction performance monitored until the fifth clutch. The tolerance of F1 neonates to starving conditions was studied monitoring the time to death of 10 neonates individually cultured in 50 mL of ASTM hard water alone. The medium was renewed every day. Life-history performance of F1 neonates was studied by culturing them individually in 100 mL of ASTM hard water at high food conditions until the release of the fourth clutch. Measured life-history traits were survival, reproduction, body length of each adult instar (including that of the adolescent instar), age at first reproduction, the size of neonates of each clutch, and the population growth rate (*r*) estimated from the age-dependent survival and reproduction rates according to the Lotka equation (Barata et al. 2001).

The second set of experiments (experiments 1 and 2) aimed to study lipid droplet changes across food and TBT treatments using the Nile red assay. In experiment 1, animals were exposed to three food regimes: starving (no food added), low food (1 × 10^5^ cells/mL *C. vulgaris*), and high food (5 × 10^5^ cells/mL *C. vulgaris*). In experiment 2, animals were exposed to two TBT concentrations (TBT L and TBT H) across low and high food levels. Exposures lasted through all the adolescent instars, and females were sampled just after their fourth molt and having released their eggs into the brood pouch (48 hr), as shown in Supplemental Material, Figure S1.

The third set of experiments aimed to determine effects of TBT L and TBT H on the dynamics of lipids, lipid droplets, and mRNA levels of selected genes across an entire adolescent intermolt cycle. Experiments were conducted only at high food levels and included five samplings: 0 hr (just after the third molt), 8 hr, 16 hr, 24 hr, and just after the fourth molt (48 hr). At each sampling, three and five replicates of 5 individuals were collected and processed for total lipid determination and mRNA gene transcription measurement, respectively, and 10 animals were processed for Nile red determination. At the 48-hr sampling period, females were de-brooded by gently flushing water into the brood pouch. Obtained eggs and de-brooded females were then collected and used for lipid and gene transcription analyses. Because of the large number of synchronized animals needed, three different independent but consecutive experiments were performed and used for lipidomic, gene transcription, and Nile red determinations, respectively.

*Nile red determination*. The Nile red stock solution was prepared in acetone and stored protected from light following Tingaud-Sequeira et al. (2011). Just before use, the working solution was prepared by diluting the stock solution to 1.5 μM in ASTM. Live individuals were then exposed to Nile red working solution in the dark for 1 hr at 20°C. After incubation, animals were placed in 100 mL ASTM for 1 min to allow clearance of Nile red residuals. Following clearance, animals were placed individually in 1.5-mL centrifuge tubes, the remaining water was removed, and samples were sonicated in 300 μL of isopropanol. The homogenized extract was then centrifuged at 10,000 × *g*. We used 200 μL of supernatant to measure Nile red fluorescence using an excitation/emission wavelength of 530/590 nm and a microplate fluorescence reader (Synergy 2, BioTek). Each treatment had one animal per sample (10 replicates in total). For each quantification and treatment, 10 blanks (animals not exposed to Nile red) were used to account for background levels of fluorescence. After exposure to Nile red, images were taken in the area surrounding the midgut for visualization of lipid droplets. Fluorescence and bright file images were obtained using a Nikon SMZ1500 microscope and a Nikon Intensilight C-HGFI with a GFP filter (EX 472/30, EM 520/35; Nikon).

*Lipidomic analyses*. Lipidomic analyses were performed as described by Gorrochategui et al. (2014), with minor modifications. Each replicate consisted of a pool of five animals that were homogenized in 500 μL phosphate-buffered saline (PBS), pH 7.4, with 2,6-di-*tert*-butyl-4-methylphenol (BHT; 0.01%) as an antioxidant. Lipid extraction was performed using a modification of Folch’s method (Folch et al. 1957). Briefly, 100 μL of the homogenized sample was mixed with 500 μL of chloroform and 250 μL of methanol. Internal standards (200 pmol) (described in Supplemental Material, Table S1) were also added. Samples were heated at 48°C overnight and dried under N_2_ the next day. Lipid extracts were solubilized in 150 μL methanol. The liquid chromatograph–mass spectrometer consisted of a Waters Aquity UPLC system connected to a Waters LCT Premier Orthogonal Accelerated Time of Flight Mass Spectrometer (Waters) operated in positive and negative electrospray ionization (ESI) mode. Full-scan spectra from 50 to 1,500 Da were obtained. Mass accuracy and reproducibility were maintained by using an independent reference spray (LockSpray; Waters). A 100-mm × 2.1-mm i.d., 1.7-μm C8 Acquity UPLC BEH (Waters) analytical column was used. Further chromatographic details of mobile phases were described by Gorrochategui et al. (2014).

Quantification was carried out using the ion chromatogram obtained for each compound using 50-mDa windows. The linear dynamic range was determined by injection of standard mixtures. Positive identification of compounds was based on the accurate mass measurement, with an error < 5 mg/L, and its LC retention time compared with that of a standard (± 2%).

A total of 116 lipids were identified and quantified by UPLC-TOF ESI-positive mode that were distributed as follows: five classes of glycerophospholipids [phosphocholine (PC) with 20 lipids, lysophosphatidylcholine (LPC) with 6 lipids, phosphatidylethanolamine (PE) with 9 lipids, phosphatidylserine (PS) with 7 lipids, and phosphatidylinositol (PI) with 3 lipids]; diacylglycerols (DG) with 20 lipids; triacylglycerols (TG) with 39 lipids; cholesterylesters (CE) with 4 lipids; and sphingolipids (SM) with 8 lipids. Glycerophospholipids, diacylglycerol, triacylglycerol, and cholesterylesters were annotated as <lipid subclass> <total fatty acyl chain length>:<total number of unsaturated bonds>. Sphingolipids were annotated as <lipid subclass> <total fatty acyl chain length>:<total number of unsaturated bonds>.

*Transcriptomic analyses*. Methods of extraction, purification, and quantification of mRNA from the studied genes and their primers follow previous procedures (Campos et al. 2013). Eight genes were selected for representation of different pathways/gene families: *EcRB, HR3, HR38, Neverland, Hb2, RXR, MET,* and *SRC*. The gene glyceraldehyde 3-phosphate dehydrogenase (*G3PDH*) was used as an internal control. For each of the genes, primers were designed using Primer Quest (IDT Technologies) and are listed in Supplemental Material, Table S2. Aliquots of 10 ng were used to quantify specific transcripts in a LightCycler® 480 real-time PCR system (Roche) using LightCycler 480 SYBR Green I Master® (Roche). Relative abundance values of all genes were calculated from the second derivative of their respective amplification curve (Cp; crossing point) values calculated by technical triplicates. Cp values of target genes were compared with the corresponding reference genes.

*Data analyses*. The effect of food rations and/or treatment or sampling period or juvenile stage on Nile red fluorescence, lipidomic profiles, mRNA abundance, and life history and physiological responses were analyzed by two-way and/or one-way analysis of variance (ANOVA). Post hoc Dunnett’s or Tukey’s tests were performed to compare exposure treatments with solvent controls. Prior to analyses, all data except survival responses were log transformed to achieve normality and variance homoscedasticity. If not indicated otherwise, significance levels were set at *p* < 0.05. Survival responses were assessed by Wilcoxon-Gehan tests. Tests were performed with IBM-SPSS statistics software, version 19. Lipidomic data were further analyzed using cluster and K-means analyses in R (R Core Team 2014) to identify clusters of lipid families similarly affected by TBT.

## Results

*Life-history consequences of exposure to TBT*. Table 1 shows a summary of life-history effects and tolerance to starvation after short-term TBT exposures in adult females (F0) and their offspring (F1) (for detailed statistical analysis results, see Supplemental Material, Tables S3 and S4). Females treated with TBT during the adolescent instar (F0) were smaller after molting (48-hr sampling point) and showed a significant decrease of the total number of their offspring. These life-history traits translated into a lower reproduction and, consequently, lower population growth rates (*r*). Adolescent females (F0) exposed to high doses of TBT during the period of egg provisioning in their ovaries also produced smaller neonates than their untreated and solvent controls or TBT L–treated counterparts, although this did not affect the tolerance of their offspring (F1) to starvation. Neonates (F1) preexposed to either concentration of TBT showed impaired survival and reduced reproduction and population growth rates, even when they grew to adulthood in a TBT-free environment (Table 1).

**Table 1:** Summary of life-history traits.

Traits	Control	Solvent control	TBT L	TBT H
Parental generation (F0)
Adult survival to 21 days (%)	90 ± 10	90 ± 10	70 ± 15	70 ± 15
Age at first reproduction (days)	9 ± 0	9 ± 0	9 ± 0	9 ± 0
Clutch size (*n*)
F0-1	10.3 ± 0.4	9.2 ± 0.6	9 ± 0.5	8.5 ± 0.4
F0-2	16.7 ± 0.6	17.1 ± 0.7	10.8 ± 0.7*	13.6 ± 1.4*
F0-3	19.8 ± 0.5	19.7 ± 0.3	15.3 ± 0.7*	15.4 ± 0.7*
F0-4	27 ± 1.0	26.6 ± 1.2	27.4 ± 1.1	27.1 ± 1.0
F0-5	27.7 ± 0.7	29.1 ± 0.7	23.7 ± 1.5*	21.1 ± 1.7*
Total offspring (*n*)	101.8 ± 1.9	101.7 ± 2.2	87.6 ± 1.8*	82.9 ± 2.6*
*r*0	0.335 ± 0.004	0.325 ± 0.009	0.313 ± 0.003*	0.304 ± 0.01*
Neonate size (μm)
N0-1	732.4 ± 6.2	723.6 ± 4.3	735 ± 6.5*	655.3 ± 4.6*
N0-2	764.5 ± 6.0	762.8 ± 10.4	766 ± 3.7	763 ± 4.8
N0-3	793.8 ± 7.7	809.6 ± 9.0	807.3 ± 5.2	801.7 ± 12
N0-4	821.4 ± 9.8	809 ± 8.7	798.7 ± 10.7	787.6 ± 8.4
N0-5	827.3 ± 10.7	834.7 ± 6.3	813.6 ± 8.2	812.4 ± 4.1
Body length (μm)
S0-1	2454.6 ± 13.9	2481.1 ± 23.4	2407.1 ± 16.2*	2305.8 ± 23.9*
S0-2	2618.2 ± 28.4	2684.9 ± 42.3	2574.3 ± 25.4*	2445.7 ± 35*
S0-3	2842.2 ± 22.6	2858.2 ± 27.5	2861.2 ± 28.9	2790.8 ± 17.1
S0-4	3145.4 ± 25.6	3133.5 ± 44.4	3108.3 ± 24.2	3,134 ± 21.3
S0-5	3275.4 ± 20.6	3248.4 ± 34.5	3274.7 ± 36.4	3239.3 ± 19.9
S0-6	3377.7 ± 20.2	3340.9 ± 29.3	3337.6 ± 25.3	3344.7 ± 14.1
First generation (F1)
N0-1 (μm)	741.1 ± 13.5	755 ± 12.2	744.1 ± 9.6*	641.7 ± 12.0*
Survival starvation (days)	6.1 ± 1.1	5.2 ± 2.4	4.5 ± 2.1	5.9 ± 1.2
Juvenile survival (%)	100 ± 0.0	100 ± 0.0	100 ± 0.0	80 ± 13.3
Adult survival to 21 days (%)	100 ± 0.0	100 ± 0.0	60 ± 16.3*	40 ± 16.3*
Age at first reproduction (days)	10.1 ± 0.1	10.1 ± 0.1	10 ± 0.0	10.9 ± 0.4
Clutch size (*n*)
F1-1	16.7 ± 0.7	17 ± 1.0	17.4 ± 1.1	14.8 ± 1.0
F1-2	20.1 ± 0.8	22.3 ± 0.7	22 ± 1.2	20.2 ± 1.6
F1-3	24.9 ± 0.7	25.1 ± 0.8	25.5 ± 1.4	22.3 ± 2.1
F1-4	27.2 ± 0.7	26.7 ± 0.6	26.8 ± 0.7*	20.3 ± 1.8*
Total offspring (*n*)	88.6 ± 2.1	91.1 ± 2.3	64.2 ± 11.2*	48.6 ± 9.9*
*r*1	0.33 ± 0.0	0.34 ± 0.0	0.28 ± 0.01*	0.23 ± 0.02*
Neonate size (μm)
N1-1	725.3 ± 9.4	717.1 ± 6.9	712.3 ± 6.2	706.6 ± 5.9
N1-2	788 ± 9.7	778.7 ± 15.1	776.3 ± 9.6	748.5 ± 17.6
N1-3	803.2 ± 8	791.7 ± 6.1	797.2 ± 4.8	810.1 ± 4.0
N1-4	866.4 ± 9.6	881.1 ± 2.6	864.4 ± 6.3	8639.2 ± 2.0
Body length (μm)
S1-1	2,656 ± 63.7	2642.3 ± 46.1	2608.7 ± 35.6	2686.8 ± 50.3
S1-2	2686.8 ± 50.3	2,656 ± 63.7	2679.6 ± 44.3	2589.6 ± 36.3
S1-3	3004.9 ± 43.6	3049.1 ± 54.4	3021.5 ± 29.1	2890.2 ± 52.4
S1-4	3202.1 ± 39	3204.4 ± 41.9	3208.5 ± 38.8	3071.9 ± 103
S1-5	3,297 ± 41.1	3337.5 ± 28.8	3340.1 ± 25.6	3258.3 ± 25.0
Values for life-history traits (mean ± SE; *n* = 10) are for adult females exposed to TBT during their adolescent instar (F0) and for their offspring (F1) that were exposed to TBT during the egg provisioning stage. Abbreviations: F, clutch size; N, neonate size; *r*, population growth rate (day^–1^); S, body length (μm). For each trait, the first number indicates the generation, and the second number refers to the brood or adult instar number. *p < 0.05 compared with solvent controls, by ANOVA and Dunnett’s post hoc test.				

**Figure 1:**
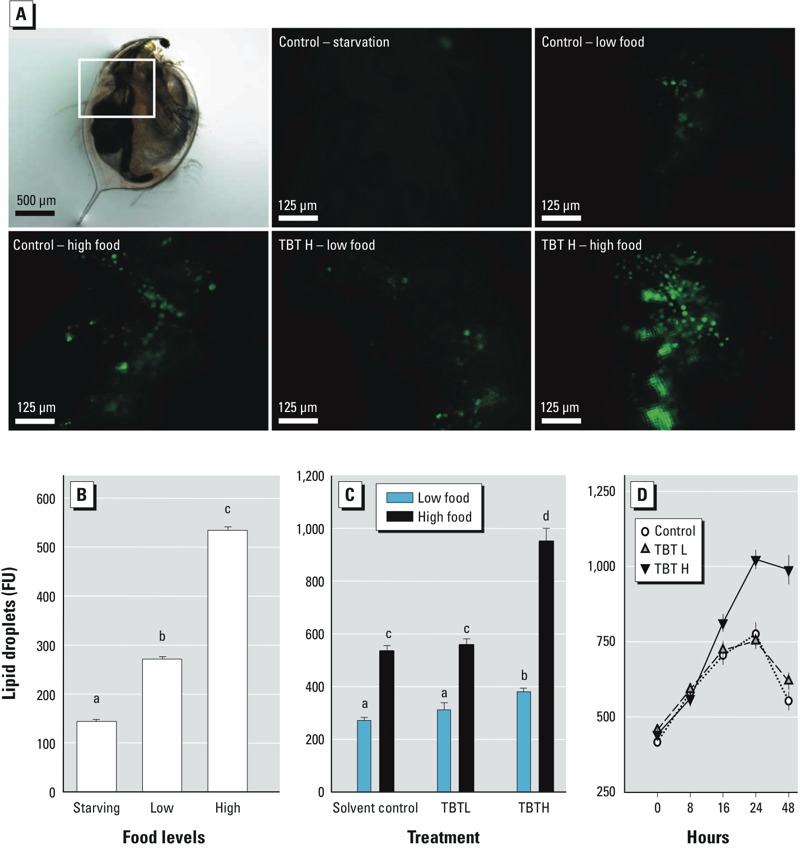
Quantitative assessment of lipid droplets in *Daphnia magna* individuals. (*A*) Lateral partial view under fluorescent microscopy of adolescent females just after molting and releasing the first brood of eggs across different food ration regimes (starving, low food, and high food) and treatments [control and TBT H (1 μg/L)]; top left, bright field microscopy image of a female, with the studied area indicated by a rectangle. Lipid droplets stained with Nile red are in green. (*B*) Nile red fluorescence [mean ± SE fluorescence units (FU); *n* = 5–10] in 48-hr females across starving, low, and high food rations, and (*C*) across TBT L and TBT H at low and high food rations. (*D*) Nile red fluorescence (mean ± SE; *n* = 5–10) measured at different time points within the adolescent instar and just after molting across TBT L and TBT H at high food rations. In *B* and* C*, different letters indicate significant (*p *< 0.05) differences among food levels or across food levels and TBT treatments, respectively, following ANOVA and Tukey’s post hoc tests. Further details are in the text.

*Nile red staining of lipid droplets*. The complex dynamics of lipid droplet dynamics in *D. magna* is summarized in Figure 1. Nile red staining showed significantly higher levels of fluorescence in females cultured at high food levels than in those reared at low food levels or starved (*F*2,27 = 144.1, *p* < 0.05; Figure 1A; the quantification of results are shown in Figure 1B). Exposure to TBT H significantly increased Nile red fluorescence in females within (*F*2,54 = 55.9, *p* < 0.05) and across (*F*2,54 = 22.7, *p* < 0.05) food levels at TBT H, such effects being more pronounced at high food levels (Figure 1A,C). The dynamics of lipid droplets during the first reproductive cycle in the presence or absence of TBT is shown in Figure 1D. In untreated females (control) or those exposed to TBT L, Nile red fluorescence increased during the intermolt period, peaked at 24 hr, and decreased just after molting and releasing of their first brood of eggs (48 hr). Exposure to TBT H significantly increased Nile red fluorescence, starting at 16 hr of exposure, reaching a maximal level at 24 hr (corresponding to twice the levels of control or TBT L samples), and remaining at this high level even after molting (48 hr). Statistical analyses showed significant (*p* < 0.05) effects of sampling period (*F*4,60 = 104.3), treatment (*F*2,60 = 31.5), and their interaction (*F*8,60 = 10.1). Whether such changes correspond to enhanced levels of TG was further studied by analyzing changes in the whole lipidome.

**Figure 2:**
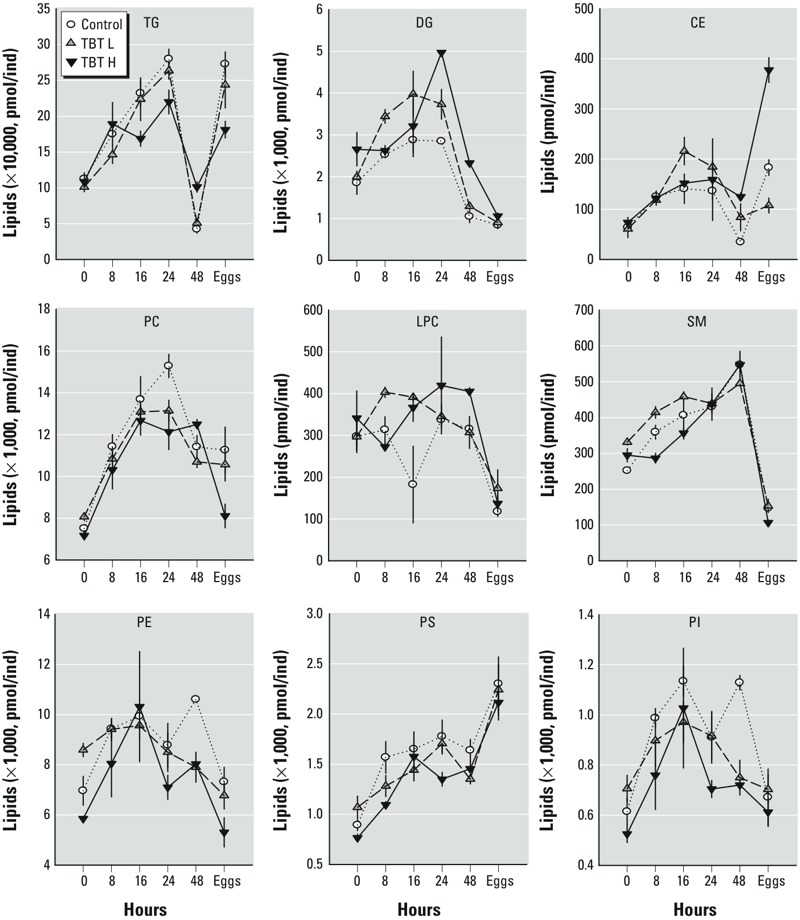
Lipidomic profiles of major lipid classes (mean ± SE; *n* = 3) in control, TBT L, and TBT H treatment groups during the adolescent instar in females at 0, 8, 16, and 24 hr, in de-brooded females just after the fourth molt (48 hr), and in eggs. Abbreviations: TG, triacylglycerols; DG, diacylglycerols; CE, cholesterylesters; PC, phosphocholines; LPC, lysophosphatidylcholine; SM, sphingolipids; PE, phosphatidylethanolamine; PS, phosphatidylserine; PI, phosphatidylinositol.

*Changes in the lipidome across TBT treatments*. Changes in the lipid content of *D. magna* juveniles, unexposed (control) or exposed to TBT L or TBT H during the adolescent instar, are shown in Figure 2 (for supporting statistics, see Supplemental Material, Table S5). The sampling time significantly affected (*p* < 0.05) the levels of the nine studied lipid classes within and across TBT concentrations (time and time × treatment effects; see Supplemental Material, Table S5). Levels of TG, DG, CE, and PC in adolescent females increased at the beginning of the instar, peaking at 16–24 hr, and decreased afterward, reaching the lowest levels just after molting in de-brooded females (48 hr). Levels of TG showed the greatest changes, increasing up to 6-fold in control females. Levels of PE, PS, PI, and SM increased during the adolescent instar, usually reaching their highest levels 24 hr after molting. In contrast, LPC showed little variation during the adolescent instar. Exposure to TBT affected levels of most lipid classes, with the exception of LPC. Levels of TG, CE, and PC were reduced by TBT exposure during the first 24 hr of the instar, but they showed increased residual levels just after molting in de-brooded females. Levels of DG in females exposed to TBT were always higher than those of controls. TBT also reduced the overall levels of lipids belonging to classes of SM, PE, PS, and PI. Unexposed eggs showed levels of TG and CE comparable to the highest levels observed in adult females, whereas PS levels were about 1.5-fold higher than those of de-brooded females just after molting. In contrast, levels of DG, PC, LPC, SM, PE, and PI were lower in eggs than in adults. Exposure to TBT reduced TG, PC, and PS levels in eggs relative to controls, and dramatically increased CE levels (Figure 2). Clustering analysis of individual lipids using K-Means identified four main clusters (see Supplemental Material, Figure S2), from which two of them (clusters 2 and 3) were particularly enriched with TG, DG, and CE. Cluster 2 included the most unsaturated TG (see Supplemental Material, Figure S3), which were mostly transferred to eggs. Within this cluster, 10 of 26 lipid species had a total fatty acyl chain length ≥ 52 and a total number of unsaturated bonds ≥ 4; thus, they could include the polyunsaturated fatty acids (PUFA) arachidonic acid (20:4) and eicosapentanoic acid (20:5) combined with two palmitic acids (16:0). Levels of these TG increased in controls through two-thirds of the instar (i.e., 24 hr), when they were mostly allocated to eggs; their levels were consequently reduced to negligible levels in de-brooded females just after molting (48 hr; see Supplemental Material, Figure S3, top). TBT H disrupted this process, making females reach peak levels earlier, maintaining high levels even after molting, and reducing the amount of these lipids allocated to eggs. Lipid profiles in eggs and females exposed to TBT L showed intermediate levels of disruption. Cluster 3 included the less unsaturated TG (see Supplemental Material, Figure S3, bottom) that were only partially (60%) transferred to eggs in control females. Exposure to TBT (either at the high or low dose) decreased the maximal attained levels of these lipids, and notably reduced their transfer to the eggs (see Supplemental Material, Figure S3, bottom).

The changes in TG, DG, CE, and PC levels in control and TBT L–exposed females correlated to the observed variations in Nile red fluorescence of lipid droplets in adult females during the instar period (compare Figure 1D with Figure 2), with Pearson correlation coefficients varying between 0.74 and 0.85 (*p* < 0.05, *n* = 10). However, for individuals exposed to TBT H, this correlation was lost for most lipid classes, except for PC and PS (*r* = 0.90 and 0.95, respectively, *n* = 5).

**Figure 3:**
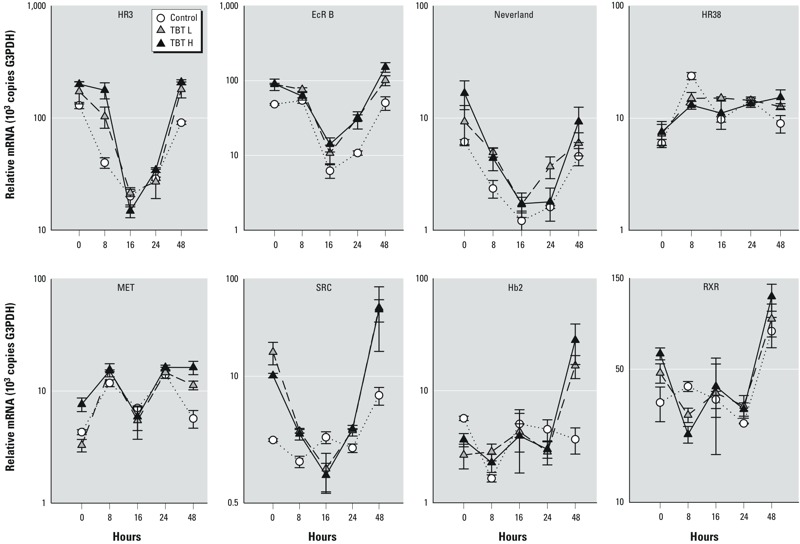
Transcription patterns (mean ± SE; *n* = 5) shown by the number of mRNA copies of the eight studied genes (*HR3*, *EcRB*, *Neverland*, *HR38*, *MET*, *SRC*, *Hb2*, and *RXR*), relative to *G3PDH*, across the adolescent instar in females exposed to TBT L (gray triangles), TBT H (black triangles), or the carrier solvent (open circles).

*Gene responses*. The profiles of mRNA abundance for the eight genes analyzed in this study during the molting/reproductive cycle in control females are shown in Figure 3. Levels of mRNA of *RXR, SRC, EcRB, HR3,* and *Neverland* genes varied during the instar, being highest at 0 and 48 hr, whereas *HR38, Hb2,* and *MET* levels remained relatively constant throughout the cycle. TBT treatments significantly increased transcript levels of seven of eight of these genes at least in some phases of the molting/reproductive cycle, including *MET* and, particularly, *Hb2*, which remained relatively constant in control conditions (Figure 3; for ANOVA results, see Supplemental Material, Table S5). Affected genes include markers of the ecdysone pathway (*EcRB, HR3, Neverland*), the juvenile hormone signaling pathway (*MET, SRC*), and the *RXR* genes.

## Discussion

Life history responses of the progeny of females exposed to TBT showed detrimental effects on fitness. Newborn neonates produced by females exposed to TBT H were smaller than those of unexposed females and suffered a higher mortality during their adulthood, which resulted in lower reproductive output and fitness. Offspring produced by females exposed to TBT L, despite being similar in size to those from unexposed females, also had lower survival during their adulthood, produced fewer eggs, and hence had a lower fitness. Eggs produced by females exposed to TBT L and TBT H also had less TG containing PUFA. These results support previous studies indicating that smaller offspring or those having low levels of PUFA are less fit than larger ones having more PUFA (Barata and Baird 1998; Gliwicz and Guisande 1992; Tessier and Consolatti 1989, 1991; Wacker and Martin-Creuzburg 2007). Exposure to TBT during a single reproductive cycle (i.e., 3–4 days) resulted in a long-lasting decrease in the females’ fitness and reproductive capacity for at least five consecutive instars. We concluded that disruptive effects of TBT on lipid metabolism reflected negatively in terms of fitness across the F0 generation and its progeny.

Changes in lipid droplet number and size, and hence in stored TG, were visualized using Nile red in *D. magna* individuals. As expected, lipid droplets were bigger and more abundant in females cultured at high food rations than in those reared at low food or starved. The complex dynamics in *Daphnia* lipid droplets described in this work reflects reported cyclic changes in TG during the reproductive cycle (Goulden and Place 1990; Tessier and Goulden 1982; Zaffagini and Zeni 1986). TG from ingested food accumulated as droplets in the animal during each intermolt interval until a few hours before molting. Upon release of the eggs into the brood pouch in adults, lipid droplets decreased as TG become allocated to the formation of the new carapace and eggs.

Lipidomic studies during the adolescent instar showed that quantitative changes in lipid droplets were highly correlated to changes in TG levels, as quantified by LC-MS, supporting the argument that lipid droplet dynamics reflect those of TG in *D. magna* individuals (Goulden and Place 1990; Tessier and Goulden 1982; Tessier et al. 1983). This correlation was lost in females exposed to TBT H. These TBT H–treated animals showed lower TG levels than controls during the first hours of the intermolt period, whereas their after-molting (48 hr) TG levels were higher than those from control or TBT L groups. In fact, lipid droplets were higher in females exposed to TBT and did not decrease in de-brooded females just after molt. This discrepancy indicates that there was less transfer of TG to egg provisioning and that TG remained stored as lipid droplets in adults. Consequently, levels of TG in the eggs of exposed females were lower than those from their nonexposed counterparts.

A lipid droplet consists of a core of neutral lipids (TG and CE) surrounded by a monolayer of phospholipid and cholesterol, into which specific proteins are embedded or peripherally associated. Little is known about the formation and metabolism of lipid droplets in *Daphnia*, but there is ample information in *Drosophila,* whose metabolism is in many aspects similar to *Daphnia* (Campos et al. 2013). *Drosophila* lipogenesis occurs in the fat cells and involves most lipid classes (Arrese and Soulages 2010). Female crustaceans convert a proportion of TG into PC to form lipovitellin, which is the major constituent of egg yolk (Lee et al. 2006). Therefore, DG, TG, CE, glycerophospholipids, and lipid droplets must be physiologically linked during the egg-provisioning period in reproductive females, which may explain their similar pattern of response in Figure 2.

Transcription levels of genes from the ecdysone and juvenile hormonal signaling pathways indicate that TBT interacts with different receptors implicated in a variety of regulatory pathways. In the present study, the receptor gene *HR3*, which is an ecdysteroid- and TBT-inducible gene in daphnids (Hannas and LeBlanc 2010; Wang et al. 2011), was up-regulated in females exposed to TBT just after molting at 0, 8, and 48 hr. This gene response corroborates the findings of Wang et al. (2011), indicating that TBT synergizes with endogenous levels of ecdysone to produce endocrine toxicity. The transcription of two additional genes involved in the ecdysone signaling hormonal pathway further showed that TBT disrupted the molt signaling pathway. Transcription patterns of the ecdysone receptor (*EcRB*) and *Neverland* genes also increased in females exposed to TBT just after molting at 0, 8, and 48 hr. The *Neverland* gene codifies for an oxygenase-like protein that plays a role in the transport and/or metabolism of cholesterol, and hence it is located upstream in the ecdysone pathway (Gilbert and Rewitz 2009; Rewitz and Gilbert 2008). In the present study, mRNA levels of *EcRB, HR3,* and *Neverland* genes were highest just after molting, which is consistent with previous reported data and reflects the natural hormonal behavior during a molt cycle (Kato et al. 2007). The three gene markers selected for the juvenile hormone signaling pathway (*MET, SRC,* and *Hb2*) increased their transcription levels in the presence of TBT just after molting at 0 hr and/or at 48 hr. There is no reported information on gene transcription responses of *MET* and *SCR* in *Daphnia*, but those reported for the hemoglobin gene (*Hb2*) were enhanced by juvenoids (Gorr et al. 2006). Results of the present study support the argument that TBT also activates the juvenile hormone receptor pathway, with the effect being greater when ecdysone levels were the highest. Nevertheless, TBT did not induce production of males in *D. magna* (data not shown), a trait characteristic of juvenoids (Wang et al. 2011), which means that TBT was interacting with the juvenile hormone signaling pathway rather than acting as a juvenoid. Transcription levels of *RXR* mRNA increased in TBT-treated females relative to the controls. We therefore conclude that TBT activates these three signaling pathways, presumably through the already proposed interaction with RXR (Wang and LeBlanc 2009; Wang et al. 2007).

## Conclusions

TBT disrupted lipid homeostasis in *D. magna* individuals by impairing the transfer of glycerophospholipids and triacylglycerols to eggs and consequently increasing the storage of lipids in lipid droplets in adults. These responses were quite similar to those reported for adipocytes in vertebrates, but their physiological consequences differed. Observed changes in the lipidome in eggs translated into smaller offspring hatched from those eggs that, later in life (during adulthood), showed impaired survival and were consequently less fit. Adult females exposed to TBT during just the adolescent instar had their reproduction and growth impaired in subsequent instars and hence were also less fit. Transcription patterns of the studied genes indicated that TBT activated the transcription of RXR receptor, as it has been reported in gastropods and vertebrates, and altered the ecdysone and juvenile hormone receptor signaling pathways as reported in other studies. Whether such effects are directly or indirectly related to the observed effects on lipid metabolism and life-history performance requires further study.

## Supplemental Material

(1 MB) PDF
