# Supplementary material for: Obesogens beyond Vertebrates: Lipid Perturbation by Tributyltin in the Crustacean Daphnia magna
Source: Environ Health Perspect. 2015 Mar 24;123(8):813–9. doi: 10.1289/ehp.1409163 (PMC4529017; doi:10.1289/ehp.1409163)
Supplement: (1 MB) PDF [file ehp.1409163.s001.acco.pdf]

Environ Health Perspect
doi: 10.1289/ehp.1409163

Note to Readers: ***EHP*** **strives to ensure that all journal content is accessible to all readers. However, some figures and Supplemental Material published in** ***EHP*** **articles may not conform to 508 standards due to the complexity of the information being presented. If you need assistance accessing journal content, please contact ehp508@niehs.nih.gov. Our staff will work with you to assess and meet your accessibility needs within 3 working days.**

Supplemental Material

Obesogens beyond Vertebrates: Lipid Perturbation by Tributyltin in the Crustacean *Daphnia magna*

Rita Jordão, Josefina Casas, Gemma Fabrias, Bruno Campos, Benjamín Piña, Marco F.L. Lemos, Amadeu M.V.M. Soares, Romà Tauler, and Carlos Barata

Table of Contents

Table S1. Internal standards used for the analysis of lipids in *D. magna*.

Table S2. Primer pairs designed.

Table S3. Repeated measurement ANOVA results on Life-History traits.

Table S4. Univariate parametric and non parametric ANOVA results on Life-History traits. ANOVA. and non parametric tests for survival, age at first reproduction, total offspring produced (Toffspring) and population growth rate (r) of females treated during the adolescent instar (parental Generation) and of offspring exposed during their egg provisioning stage (F1 Generation).

Table S5. Two way ANOVA on lipid classes and mRNA gene responses.

Figure S1. Diagram of the test protocol used to exposed *D. magna* and measure changes in lipids and lipid droplets. *D .magna* individuals were fed on high food during their growth until 2/3 of their third juvenile instar and then exposed to the studied food treatments, TBT and its solvent carrier during a little bite more than the adolescent instar.

Figure S2. Heat map and hierarchical clustering (K-Means) of the quantified lipid groups in *Daphnia magna* juveniles along the studied period of the adolescent instar in control (C), TBTL

(L) and TBT H (H) treatments. High and low lipid levels are in red and blue and those in yellow unchanged.

Figure S3. Mean levels of individual TG differentiated in clusters 2 (upper) and 3 (lower graph panel) depicted in Supplemental Material, Fig S2 for control (C), TBTL and TBTH treatments along the adolescent instar at 0, 8, 16 and 24 h and in adults just after the forth molt without eggs (48 h) and in eggs. Lipid compounds are depicted in the legends.

Table S1. Internal standards used for the analysis of lipids in *D. magna*.

Family	Name	Abbreviation	Molecular Formula	Molecular weight	Exact mass
Neutral glycerolipids	Triheptadecanoate 1,2,3-triheptadecanoyl-glycerol	TAGC51:0	$C_{54}H_{104}O_6$	849.4	848.783
	1,3 (d5)-diheptadecanoyl-glycerol	DAG C34:0	$C_{37}H_{67}D_5O_5$	601.995	601.569
	1-heptadecanoyl-rac-glycerol	MAG C17:0	$C_{20}H_{40}O_4$	344.529	344.293
Sterols	cholest-5-en-3ß-yl heptadecanoate	CE C17:0	$C_{44}H_{81}O_2N$	639.089	638.6
Phospholipids	1-hexadecanoyl(d31)-2-(9Z-octadecenoyl)-sn-glycero-3-phosphocholine	d31PC 34:1	$C_{42}H_{51}D_{31}NO_8P$	791.267	790.772
	1-hexadecanoyl(d31)-2-(9Z-octadecenoyl)-sn-glycero-3-phosphoethanolamine	d31PE 34:1	$C_{39}H_{45}D_{31}NO_8P$	749.187	748.725
	1-hexadecanoyl(d31)-2-(9Z-octadecenoyl)-sn-glycero-3-[phospho-L-serine]	d31PS 34:1	$C_{40}H_{45}D_{31}NO_{10}P$	815.179	814.697
Lysophospholipids	1-(10Z-heptadecenoyl)-sn-glycero-3-phosphoethanolamine	LPE C17:1	$C_{22}H_{44}NO_7P$	465.561	465.286
	1-(10Z-heptadecenoyl)-sn-glycero-3-phospho-L-serine	LPS C17:1	$C_{23}H_{44}NO_9P$	531.552	531.257
	1-heptadecanoyl-sn-glycero-3-phosphate	LPA C17:0	$C_{20}H_{41}O_7P$	446.491	446.241
	1-heptadecanoyl-sn-glycero-3-phosphocholine	LPC C17:0	$C_{25}H_{53}NO_7P$	509.657	509.348
Sphingolipids	N-lauroyl-D-*erythro*-spingosylphosphorylcholine	SM C12:0	$C_{35}H_{71}N_2O_6P$	646.922	646.505

Table S2. Primer pairs designed.

Genes	Accession Number	Forward	Reverse	Amplicon size
G3PDH	AJ292555	ACGAGACCCGAAAAACATTCC	CAATGTGAGCATGGGCCTTT	100
EcRB	AB274824	CACCACAACCAACTGCATTTAC	CCATTAATGTCAAGATCCCACA	81
HR3	FJ755466	AAGGTCGAGGATGAAGTGCG	AAAGACGCTACTATCGGGCG	81
HR38	KM982449	AGTGAGCGGAGTTCTGGCAG	CGTGACATATAACCCGGAAGC	81
Neverland	KM893860	CAAATGAGGGCAATACGCGT	GATGCTCTCGGCGAGAACAT	81
Hb2	AB021136	CCCAGGTTCTTTTCCGCCTTC	CGGATTGAGGAACATCGGC	81
RXR	DQ530508	GTGTCGAGTGCAAGGACGAG	CCCATTCAACCAACTGGAAAA	100
SRC	AB698070	TACTAGGCGTCTTGCTGAATGAA	CCATAATTTGCAAGGCTCCG	81
MET	AB698069	CAAACAGCCAGAGATTACCGG	GCACTGTTGGTTCCAGCATTC	81

Primers has been obtained from existing sequences used for the analyses of gene transcriptomic changes.

Table S3. Repeated measurement ANOVA results on Life-History traits.

**Parental Generation**			**F1 Generation**		
**Clutch size**	**df**	**F**	**Clutch size**	**df**	**F**
Brood	1,26	851.1**	Brood	1,25	213.4**
Treatment	3,26	15.1**	Treatment	3,25	3.2*
Brood x Treatment	3,26	4.5*	Brood x Treatment	3,25	2.1 ns
**Neonate size**	**df**	**F**	**Neonate size**	**df**	**F**
Brood	1,26	368.4**	Brood	1,25	357.6**
Treatment	3,26	12.7**	Treatment	3,25	0.6 ns
Brood x Treatment	3,26	5.8**	Brood x Treatment	3,25	1.4 ns
**Body length**	**df**	**F**	**Body size**	**df**	**F**
Brood	1,28	3171.1**	Brood	1,25	839.4**
Treatment	3,28	3.9*	Treatment	3,25	0.2 ns
Brood x Treatment	3,28	8.3**	Brood x Treatment	3,25	0.4 ns

Brood number was used as a repeated measure and treatment as a fixed factor for clutch and neonate size and body length across broods of females treated during the adolescent instar and of offspring exposed during their egg provisioning stage. ns p>.05; * .05<p<.01, ** .01<p<.001, ***p< .001

Table S4. Univariate parametric and non parametric ANOVA results on Life-History traits. ANOVA. and non parametric tests for survival, age at first reproduction, total offspring produced (Toffspring) and population growth rate (r) of females treated during the adolescent instar (parental Generation) and of offspring exposed during their egg provisioning stage (F1 Generation).

**Parental Generation**				**F1 Generation**			
		**df**	**Statistic**			**df**	**Statistic**
Survival adult	Wilcoxon (Gehan) Statistic	3	2.4 ns	Survival starvation	Chi-Square	3	4.3 ns
				Survival juvenile	Wilcoxon (Gehan) Statistic	3	6.1 ns
				Survival adult	Wilcoxon (Gehan) Statistic	3	13.7**
Age	Kruskal-Wallis	3	0 ns	Age	Kruskal-Wallis	3	6.4 ns
Toffspring	ANOVA	3,31	19.9**	Toffspring	ANOVA	3,36	7.1**
r	ANOVA	3,36	4.6**	r	ANOVA	3,36	7.5**

Analyses were performed on survival, age at first reproduction, total offspring produced (Toffspring) and population growth rate (r) of females treated during the adolescent instar (parental Generation) and of offspring exposed during their egg provisioning stage (F1 Generation). ns p>.05; * .05<p<.01, ** .01<p<.001, ***p< .001

Table S5. Two way ANOVA on lipid classes and mRNA gene responses.

	Treatment		Time		Treatment x Time	
	**df**	**F**	**df**	**F**	**df**	**F**
**Lipids**						
PC	2,36	6.5**	5,36	43.6***	10,36	2.4*
LPC	2,33	2.1 ns	5,33	22.4***	10,33	1.0 ns
PE	2,36	8.4**	5,36	10.2***	10,36	1.4 ns
PS	2,36	6.5**	5,36	38.8***	10,36	1.5 ns
PI	2,36	7.1**	5,36	10.0***	10,36	1.0 ns
TG	2,36	0.6 ns	5,36	112.2***	10,36	7.9***
DG	2,36	14.9***	5,36	69.8***	10,36	2.9**
CE	2,36	12.2***	5,36	25.3***	10,36	4.3**
SM	2,36	10.1***	5,36	169.3***	10,36	3.3**
**Genes**						
HR3	2,47	4.7*	4,47	30.0***	8,47	3.1**
EcR B	2,47	17.1***	4,47	26.8***	8,47	26.1***
Neverland	2,48	8.2**	4,48	20.2***	8,48	1.6 ns
HR38	2,48	0.5 ns	4,48	14.9 ***	8,48	1.9 ns
MET	2,47	6.6**	4,47	29.5***	8,47	3.1**
SCR	2,47	18.8***	4,47	78.3***	8,47	9.9***
Hb2	2,48	2.7 ns	4,48	23.6***	8,48	8.1***
RXR	2,48	1.5 ns	4,48	42.7***	8,48	3.2**

ANOVA results (Fisher's coefficient, F, degrees of freedom, df) comparing the response of lipid classes and mRNA gene responses across TBT treatments along the adolescent instar, in de-brooded females just alter the forth molt and in eggs. For mRNA levels treatments did not included eggs. ns P>0.05; * 0.05<P<0.01, ** 0.01<P<0.001, ***P< 0.001

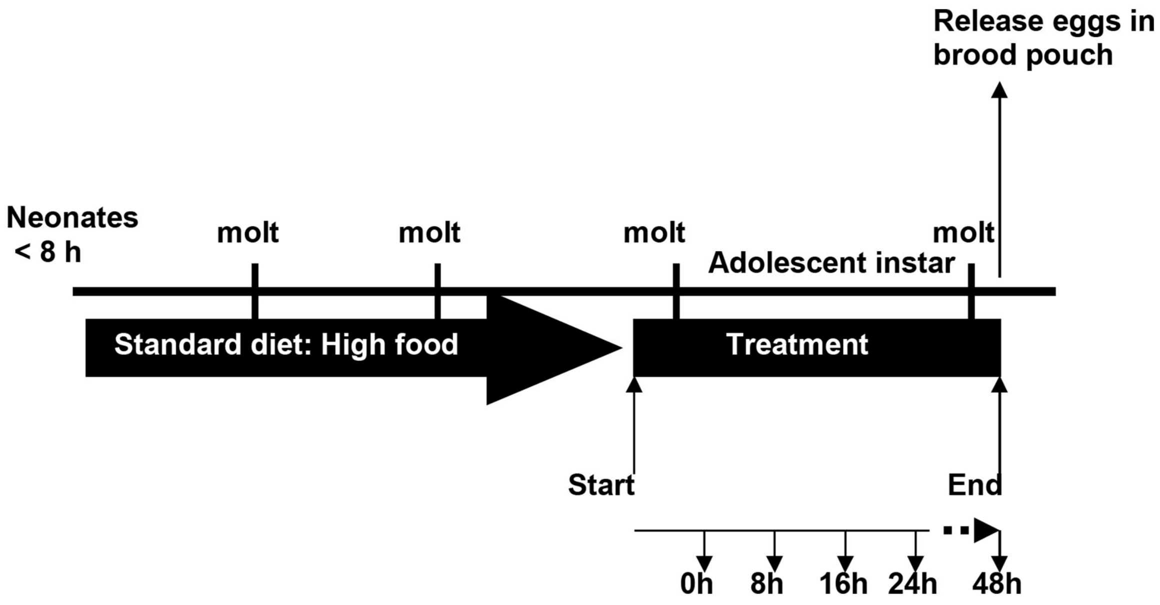

Figure S1. Diagram of the test protocol used to exposed *D. magna* and measure changes in lipids and lipid droplets. *D .magna* individuals were fed on high food during their growth until 2/3 of their third juvenile instar and then exposed to the studied food treatments, TBT and its solvent carrier during a little bite more than the adolescent instar.

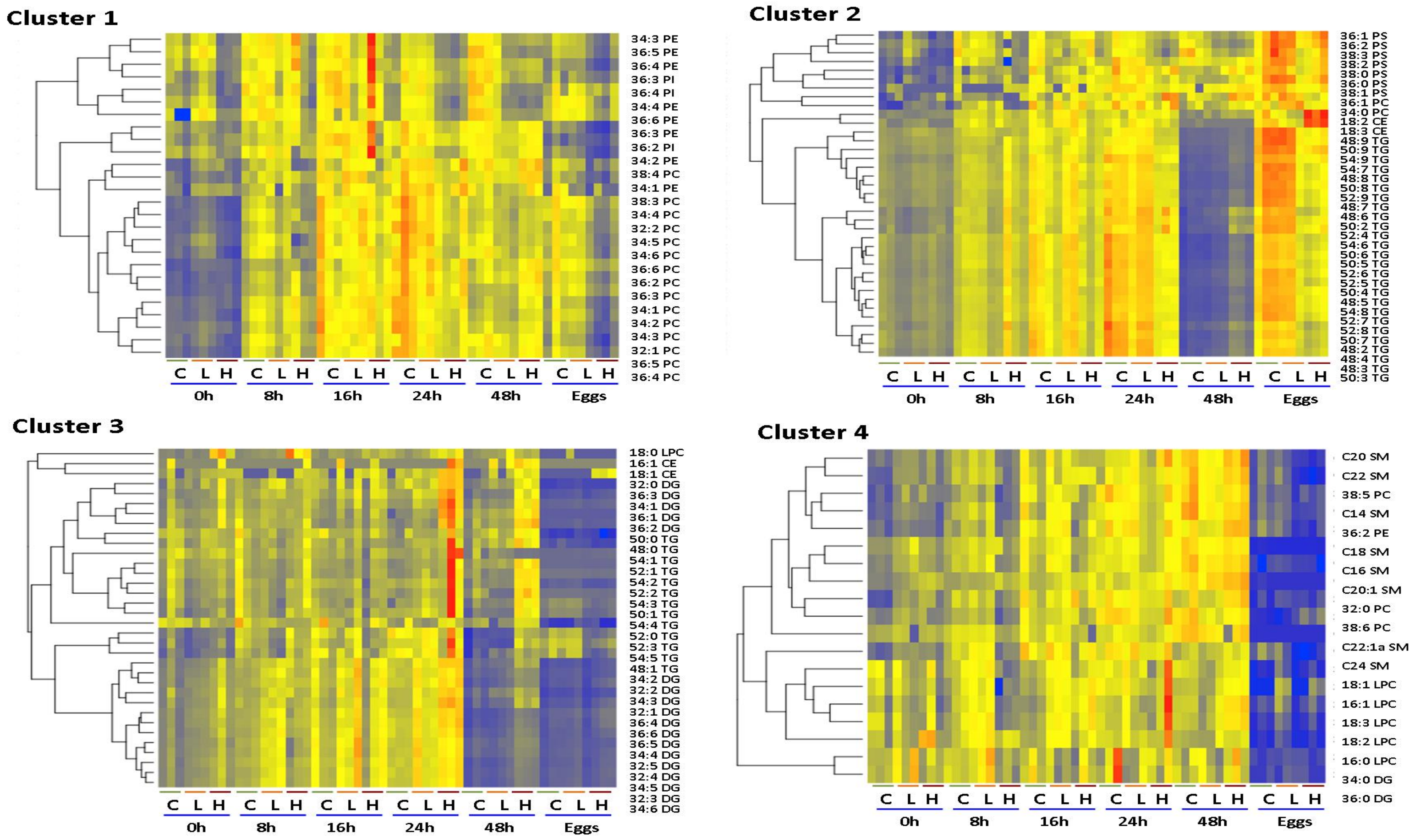

Figure S2. Heat map and hierarchical clustering (K-Means) of the quantified lipid groups in *Daphnia magna* juveniles along the studied period of the adolescent instar in control (C), TBTL (L) and TBT H (H) treatments. High and low lipid levels are in red and blue and those in yellow unchanged.

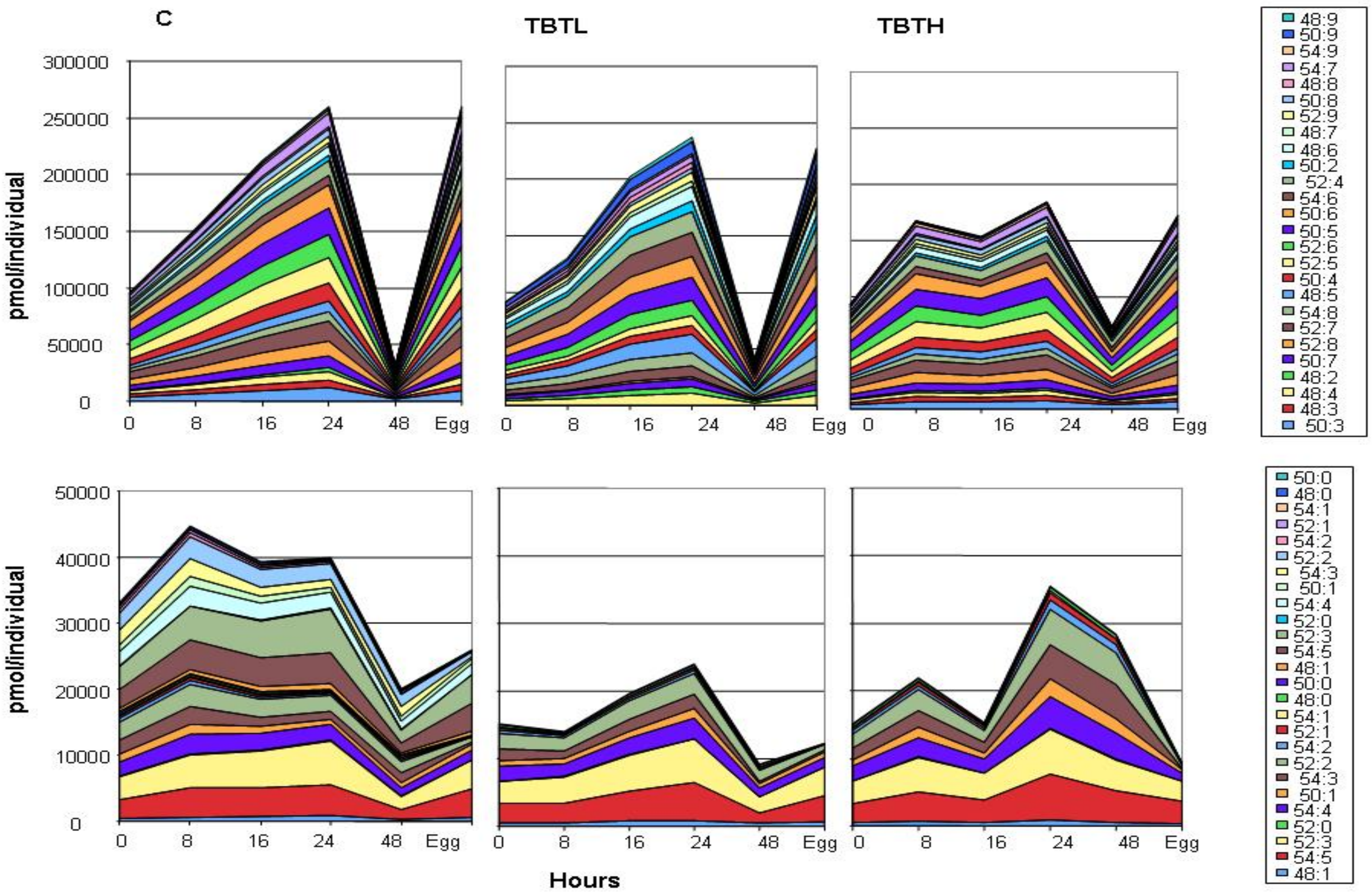

Figure S3. Mean levels of individual TG differentiated in clusters 2 (upper) and 3 (lower graph panel) depicted in Supplemental Material, Fig S2 for control (C), TBTL and TBTH treatments along the adolescent instar at 0, 8, 16 and 24 h and in adults just after the forth molt without eggs (48 h) and in eggs. Lipid compounds are depicted in the legends.
